# Cyclin Y-mediated transcript profiling reveals several important functional pathways regulated by Cyclin Y in hippocampal neurons

**DOI:** 10.1371/journal.pone.0172547

**Published:** 2017-02-27

**Authors:** I-Seul Joe, Jong-Hwan Kim, Hanna Kim, Jung-Hwa Hong, Mirang Kim, Mikyoung Park

**Affiliations:** 1 Center for Functional Connectomics, Korea Institute of Science and Technology, Seoul, South Korea; 2 Personalized Genomic Medicine Research Center, Korea Research Institute of Bioscience and Biotechnology, Daejeon, South Korea; 3 Department of Functional Genomics, Korea University of Science and Technology, Daejeon, South Korea; 4 Department of Life Sciences, Korea University, Seoul, South Korea; 5 Department of Neuroscience, Korea University of Science and Technology, Daejeon, South Korea; Western University of Health Sciences, UNITED STATES

## Abstract

Cyclin Y (CCNY), which is a cyclin protein known to play a role in cell division, is unexpectedly and thus interestingly expressed in non-proliferating neuronal cells. There have been only a few studies reporting the neuronal functions of CCNY in synapse remodeling and hippocampal long-term potentiation. Therefore, we here provide global and comprehensive information on the putative functions of CCNY in biological and functional pathways in neuronal systems. We adopted high-throughput RNA-sequencing technology for analyzing transcriptomes regulated by CCNY and utilized bioinformatics for identifying putative molecules, biological processes, and functional pathways that are possibly connected to CCNY functions in hippocampal neuronal cells of rats. We revealed that several enriched annotation terms and pathways associated with CCNY expression within neurons, including apoptosis, learning or memory, synaptic plasticity, actin cytoskeleton, focal adhesion, extracellular matrix-receptor interaction and chemokine signaling pathway are targeted by CCNY. In addition, the mRNA levels of some genes enriched for those annotation terms and pathways or genes reported to be altered in Alzheimer’s disease mouse model were further validated by quantitative real-time PCR in hippocampal neuronal cells. The present study provides an excellent resource for future investigations of CCNY functions in neuronal systems.

## Introduction

Cyclin Y (CCNY) is one of the members of the cyclin family that has been known to regulate cell division in proliferating cells [1–3]. CCNY was originally identified as an interacting protein of the cyclin-dependent kinase CDK14/PFTK1 via a yeast two-hybrid screen [4]. Its role has been investigated in the field of cancer biology by showing that CCNY regulates glioma and lung cancer cell proliferation [5, 6]. In addition, CCNY played an essential role in the maintenance of mammary stem/progenitor cell properties [7] and the control of adipogenesis and lipid production [8]. Furthermore, CCNY was a key factor for the development of Drosophila, including larval growth, pupal development and metamorphosis [2].

Interestingly, CCNY has been shown to play roles in non-dividing neuronal cells. Role of CCNY in the nervous system was first described in *C*. *elegans* as a regulator for synapse formation and elimination [9, 10], and it was also found in the mammalian nervous system as a negative regulator for hippocampal long-term potentiation (LTP) [11], the most widely studied cellular basis of learning and memory [12–15]. Investigating the function of CCNY in the non-proliferating neuronal cells is intriguing since CCNY has been generally known for its role in proliferating cells. Although a few studies reported on the role of CCNY in the nervous system [9–11], the mechanistic and signaling information on how CCNY functions in the brain remains mostly unknown. In this study, we provide candidate molecules, biological processes and functional signaling pathways that might be regulated by CCNY, a relatively novel molecule whose function has been rarely investigated.

RNA sequencing (RNA-seq), which is a recent revolutionary tool providing an accurate and precise measurement of transcript levels, has been widely applied for systematic, comprehensive, and global analysis of transcriptome in various species [16–18]. This next-generation high-throughput sequencing technology has provided an unbiased approach for investigating pathophysiology of neurodegenerative diseases [19–22]. In this study, the RNA-seq technique, bioinformatics, and quantitative real-time PCR (qRT-PCR) have been adopted to extract molecular profiles that are regulated by CCNY in hippocampal neuronal cells and provide invaluable information on putative biological processes, molecular functions and functional signaling pathways that CCNY may be involved in hippocampal neuronal system. The extensive and essential resources provided in the present study will serve as a platform for future investigations of CCNY function in neuronal systems.

## Materials and methods

### Cell culture

HEK 293T cells were grown in DMEM (HyClone) supplemented with 10% fetal bovine serum. Hippocampal neuron cultures were prepared from E18 Sprague-Dawley (SD) rat embryos and maintained for 14–21 days *in vitro* (DIV) [11]. All experiments handling animals and their embryos were performed in accordance with the guidelines and regulations of the Korea Institute of Science and Technology (KIST). All experimental protocols were approved by the KIST Institutional Animal Care and Use Committee (IACUC; approval number 2016–065).

### DNA constructs

The same constructs from our previous study [11] were used for CCNY-WT-EGFP, FUGW-CCNY-WT, and FUGW-CCNY-shRNA.

### Immunocytochemistry

For staining endogenous PSD-95, hippocampal neurons on coverslips were fixed with 4% paraformaldehyde/4% sucrose in phosphate-buffered saline (PBS) for 15–20 min at room temperature and permeated with 0.1% TritonX-100 in PBS for 10 min at room temperature. Neurons were then incubated with mouse anti-PSD-95 (MA1-046, Thermo fisher scientific, 1:200) in PBS containing 5% normal donkey serum for 1 hr at room temperature. Anti-mouse Cy3-conjugated secondary antibody (1:300) was applied for 45 min at room temperature. Coverslips were then mounted on slide glasses for imaging.

### Production of lentivirus

Lentivirus expressing EGFP, CCNY-WT-EGFP or CCNY-shRNA-EGFP was generated as described in our previous study [11]. Briefly, lentiviral vector FUGW, FUGW harboring CCNY-WT or CCNY-shRNA, the packaging vector Δ8.9, and VSVG envelope glycoprotein vector were co-transfected into HEK 293T cells using X-tremeGENE HP DNA transfection reagent (Roche). Thirty six to 48 hours after transfection, supernatants containing the lentivirus were harvested, aliquoted, and stored at −80°C.

### Sample preparation for RNA-seq

Cultured hippocampal neurons were infected with lentivirus expressing EGFP, CCNY-WT-EGFP or CCNY-shRNA-EGFP at DIV5-6, and the neuronal cell lysates were harvested at DIV14 for total RNA isolation and subsequent RNA-seq.

### RNA extraction, cDNA library construction, RNA-Seq and data analysis

RNA-seq was performed as described [23]. Total RNA was isolated using the RNeasy kit (Qiagen, Valencia, CA), the RNA-seq library was prepared using the TruSeq RNA Sample Prep Kit (Illumina, San Diego, CA, USA) and the sequencing was performed based on Illumina NextSeq500 platform to generate 150-bp paired-end reads. The sequenced reads were mapped to the Rat genome (rn4) using TopHat 2, and the gene expression levels were calculated using Cufflinks [24, 25]. The cuffdiff module in the cufflinks package was used to select differentially expressed genes (DEGs) from the RNA-seq data which cover the total 17,066 genes. Meanwhile, the FPKM value of each gene was floored to 1, and log2-transformed for further analysis. Heat maps were constructed using Mev [26]. Statistical analyses and graph construction were performed using R (v. 3.1.0) and PYTHON (v. 2.7.6).

### Bioinformatic analysis

To extract the over-represented (enriched) biological annotation terms and pathways from DEGs, the Database for Annotation, Visualization and Integrated Discovery (DAVID) Bioinformatics Resources v6.7 (https://david.ncifcrf.gov) [27, 28] was applied for the Gene ontology (GO) analysis on the basis of three categories, including biological process, cellular component and molecular function and also for the Kyoto Encyclopedia of Genes and Genomes (KEGG) enrichment analysis [29, 30]. The enriched GO terms and enriched KEGG pathways were first identified based on the uncorrected P-values, and only the terms and pathways that are likely related to mainly neuronal functions and broadly cell proliferation were represented as figures (S2–S4 Figs). Some of the identified terms and pathways from the S2–S4 Figs are also presented as Tables, including the individual genes belonging to each term and pathway (S1 and S2 Tables). RNA-seq data were closely investigated for all individual genes belonging to the terms and pathways listed in S1 and S2 Tables. Then, genes showing apparent disparity in the transcript levels between control, CCNY-WT overexpression, and CCNY knockdown samples were chosen for quantitative real-time PCR (qRT-PCR) validations. Only the genes showing statistical significance between the samples of control, CCNY-WT overexpression, and CCNY knockdown in mRNA levels quantified by qRT-PCR were presented as data in this study. The result of Cxcl1 gene showing no significance between the samples was included in the data as a control, which is consistent with the reported study [31].

### RNA-seq data access

RNA-seq data have been deposited in the NCBI Gene Expression Omnibus (GEO) under the accession number GSE84850.

### Quantitative Real-Time PCR (qRT-PCR) and analysis

Cultured hippocampal neurons infected with lentivirus expressing EGFP, CCNY-WT-EGFP or CCNY-shRNA-EGFP for 7–8 days were harvested. Total RNA was extracted using RNAiso Plus (TaKaRa, Japan) according to the manufacturer’s instructions and was reverse transcribed into cDNA using the the PrimeScript II 1^st^ strand cDNA synthesis kit (TaKaRa, Japan). The qRT-PCR was performed using Power SYBR Green PCR Master Mix (Thermo fisher scientific). The reaction mixture contained 0.5 μl of cDNA corresponding to 75 ng of total RNA, 150 nM of each gene-specific primers and 2x Power SYBR Green PCR Master Mix in a total volume of 20 μl. The cycling parameters of StepOnePlus Real-Time PCR System (Applied Biosystems) were as follows: 95°C for 10 min, followed by 40 cycles of 95°C for 15 sec and 60°C for 1 min. Relative expression level was calculated according to the 2^−ΔΔCT^ algorithm based on the expression of glyceraldehyde 3-phosphate dehydrogenase (GAPDH) gene, which did not show differential expression among samples in the study. Experiments were independently performed 3 times with duplicates each time. Primer sets used for qRT-PCR are listed in S3 Table.

### Immunoblot analysis

Equal amounts of protein denatured in SDS sample buffer were applied to SDS-PAGE, transferred onto a PVDF membrane, and applied to immunoblot reactions. Protein bands were visualized by a chemiluminescence method (Millipore or Thermo fisher scientific) and an imaging documentation system (ImageQuant LAS 4000, GE healthcare). Primary antibodies against CCNY (Proteintech group), GFP (Roche) or β-tubulin (Abcam) were used.

## Results

### Experimental model system for RNA-seq based transcriptome analysis

**Fig 1:**
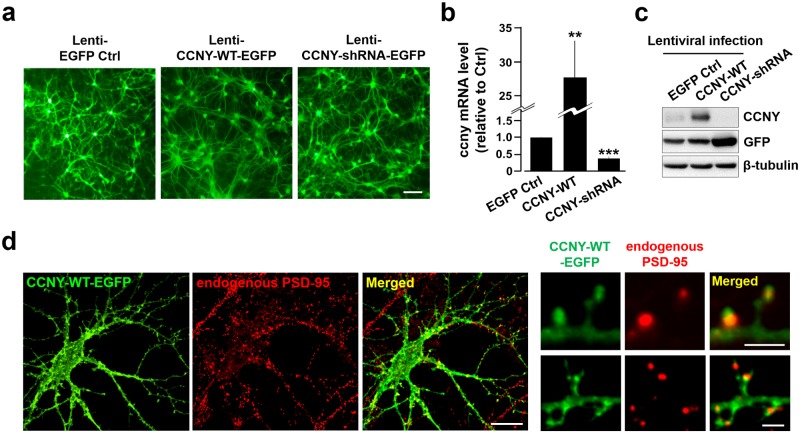
Experimental model systems for RNA-seq based transcriptome analysis. (**a**) Cultured hippocampal neurons expressing EGFP (Lenti-EGFP Ctrl), CCNY-WT-EGFP (Lenti-CCNY-WT-EGFP) or CCNY-shRNA-EGFP (Lenti-CCNY-shRNA-EGFP) via lentiviral expression system. (**b**,**c**) Relative levels of mRNA (**b**) and protein (**c**) of CCNY from the neurons infected with lentivirus expressing EGFP, CCNY-WT-EGFP or CCNY-shRNA-EGFP. n = 5 from 3 independent experiments. **p<0.01 relative to control, ***p<0.005 relative to control, student’s *t* test. Refer to the S9 Fig for the full-length blots of (**c**). (**d**) CCNY-WT exists in the spines near the endogenous PDS-95 in cultured hippocampal neurons, supporting the value of the study on systematic analysis for putative neuronal functions of CCNY. Scale bars, 20 and 2 μm for the whole neuronal and enlarged images, respectively.

CCNY is unexpectedly expressed in non-proliferating neuronal cells [11]. However, there are only few reports on the function of CCNY in neuronal systems, which include its regulation of synapse remodeling and hippocampal LTP [9–11]. Therefore, further investigation on the neuronal functions of CCNY is indispensable. For this aim, we first searched for systematic information of gene sets that are possibly regulated by CCNY in the hippocampal neurons; the region of hippocampus was chosen based on the previous report on the function of CCNY in hippocampal LTP [11]. We lentivirally overexpressed or knockdowned CCNY in the primary cultured hippocampal neurons (Fig 1a) and confirmed that CCNY mRNA (Fig 1b; EGFP control, 1.0 ± 0.012; CCNY-WT, 27.87 ± 5.365; CCNY-shRNA, 0.37 ± 0.063) and protein levels (Fig 1c) were significantly enhanced and reduced in neurons overexpressing and knocking down CCNY, respectively. Consistent with the previous report showing the existence of CCNY in the postsynaptic subcellular fraction [11], CCNY wild-type (CCNY-WT) localizes adjacent to the endogenous postsynaptic density protein-95 (PSD-95) (Fig 1d), supporting the value of systematic analysis on putative neuronal functions of CCNY.

### Up- and down-regulated genes by CCNY in the hippocampal neurons

**Fig 2:**
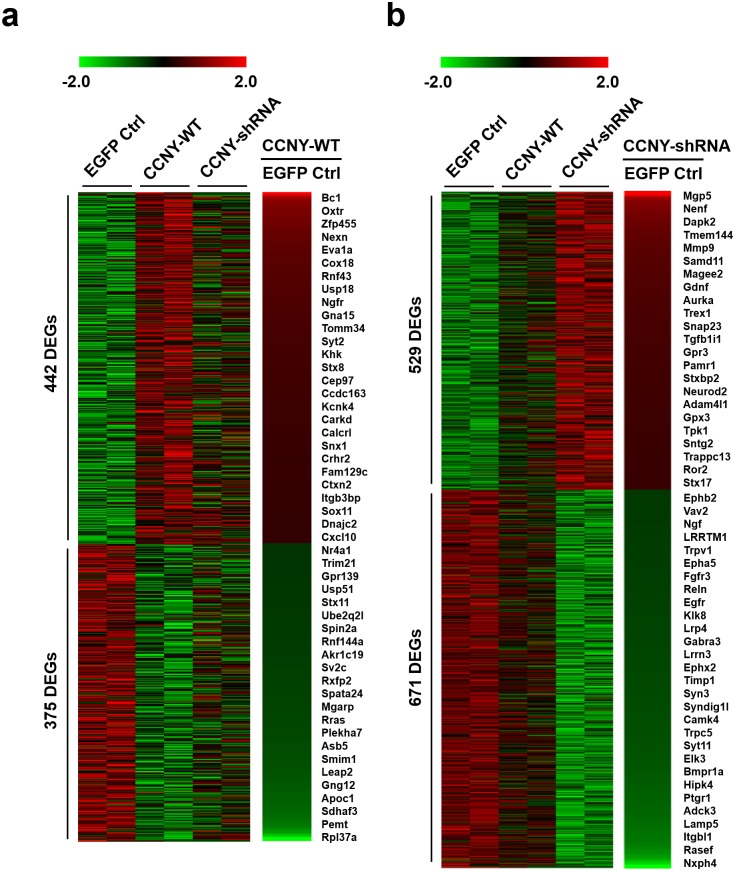
Up- and down-regulated genes by CCNY in the hippocampal neurons. (**a**,**b**) Heatmaps of differentially expressed genes (DEGs) for up- or down-regulated genes by CCNY overexpression (**a**) or knockdown (**b**). Selected gene names are listed on the right side of the heatmaps. Red indicates up-regulated gene expression level, whereas green indicates down-regulated gene expression level. The values of log_2_FPKM were normalized to the value ranges from minimum -2.0 to maximum +2.0.

We next aimed to obtain the profile of genes that are regulated by CCNY in hippocampal neurons and thus performed RNA-seq from each hippocampal neuronal samples that are CCNY overexpressed or CCNY knocked down. Gene expression level was calculated as the fragments per kilobase of transcript per million fragments mapped (FPKM). We found that the log_2_FPKM of CCNY is enhanced in the CCNY overexpressed neurons whereas it is reduced in the CCNY knocked down neurons (S1a Fig). The fold change (fc) was calculated by subtracting the FPKM value of EGFP control from the FPKM value of CCNY-EGFP overexpression or CCNY shRNA-mediated knockdown samples. Differentially expressed genes (DEGs) were selected by setting |log_2_fc| ≥ 0.3 and |log_2_fc| ≥ 0.33 for CCNY overexpression and knockdown, respectively. With these criteria, the total of 442 up-regulated and 375 down-regulated DEGs in CCNY-WT-overexpressing neurons and 529 up-regulated and 671 down-regulated DEGs in CCNY-shRNA-knockdown neurons were identified (S1b Fig), and the differential expression profiles demonstrating up- and down-regulations by CCNY overexpression (Fig 2a) or knockdown (Fig 2b) were displayed.

### CCNY-mediated regulation of biological processes, including apoptosis and learning or memory

**Fig 3:**
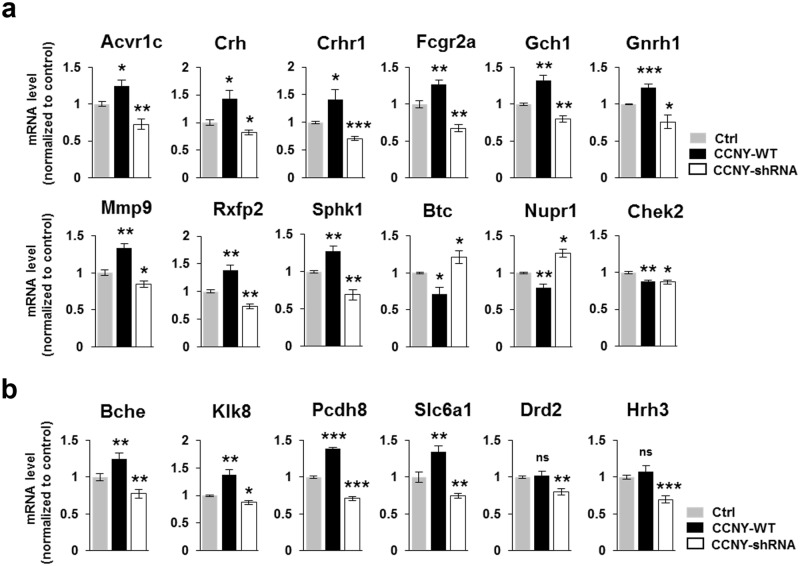
CCNY affects the biological processes related to apoptosis, and learning or memory in hippocampal neurons. (**a**,**b**) Validation of several genes in GO terms regulated by CCNY. qRT-PCR validations of several genes belonging to the term “(positive) regulation of apoptosis” in up-regulated GO terms by CCNY overexpression or down-regulated GO terms by CCNY knockdown (**a**), the term “learning or memory” in down-regulated GO terms by CCNY knockdown and the term “regulation of synaptic plasticity” in down-regulated GO terms by CCNY knockdown (**b**). Refer the S1 Table.

To better understand the function of CCNY in neuronal cells, we used the Database for Annotation, Visualization and Integrated Discovery (DAVID) functional annotation tool for conducting the Gene ontology (GO) analysis on the basis of three categories, including biological process, cellular component, and molecular function [32] from each sets of DEGs shown in Fig 2. Positive regulation of apoptosis in biological process, extracellular region/space in cellular component, hormone activity, cytokine activity, pattern binding, polysaccharide binding, glycosaminoglycan binding, growth factor activity, protein dimerization activity, voltage-gated ion channel activity, peptide receptor activity, metal ion transmembrane transporter activity, and carbohydrate binding in molecular function were selected as the GO terms that are significantly up-regulated by CCNY overexpression while down-regulated by CCNY knockdown (S2a Fig). In addition, regulation of apoptosis and regulation of cell proliferation in biological process and extracellular space in cellular component were selected as the GO terms that are both significantly up-regulated by CCNY knockdown and down-regulated by CCNY overexpression (S2b Fig). Using qRT-PCR, we further revealed that the mRNA expression levels of genes such as Acvr1c, Crh, Crhr1, Fcgr2a, Gch1, Gnrh1, Mmp9, Rxfp2, Sphk1, Btc, Nupr1, and Chek2 belonging to the (positive) regulation of apoptosis in our analysis (S1 Table) were regulated by CCNY (Fig 3a). These data suggest a role of CCNY in apoptosis, which is highly plausible based on the previous reports showing the regulation of synaptic plasticity such as LTP and long-term depression (LTD) by a signaling pathway involving apoptotic molecules [33, 34].

In addition to the terms mentioned above, the GO terms related to neuronal functions, including learning or memory, regulation of synaptic plasticity, neuron development, regulation of neurological system process, and/or positive regulation of glutamatergic synaptic transmission in biological process were also significantly enriched in the down-regulated DEGs by CCNY knockdown or overexpression (S2a Fig), which could be predicted from the previous reports showing the CCNY functions in synapse formation, elimination and plasticity [9–11]. Several genes (Bche, Klk8, Pcdh8, Slc6a1, Drd2, and Hrh3) belonging to the terms such as learning or memory, and synaptic plasticity (S1 Table) were further validated to be regulated by CCNY using qRT-PCR (Fig 3b).

### CCNY-mediated regulation of pathways for the regulation of actin cytoskeleton, focal adhesion, and Extracellular Matrix (ECM)-receptor interaction

We next carried out the Kyoto encyclopedia of genes and genomes (KEGG) pathway enrichment analysis [29, 30] to identify enriched metabolic or signaling pathways in each sets of DEGs shown in Fig 2. Regulation of actin cytoskeleton and chemokine signaling pathway were selected as the KEGG pathways that are up-regulated by CCNY overexpression while down-regulated by CCNY knockdown (S3a Fig). In addition, neuroactive ligand-receptor interaction, calcium signaling pathway, cytokine-cytokine receptor interaction, focal adhesion, ECM-receptor interaction, axon guidance, melanoma, antigen processing and presentation were identified as the KEGG pathways that are significantly down-regulated by CCNY knockdown (S3a Fig). DNA replication, systemic lupus erythematosus, and SNARE interactions in vesicular transport were significantly enriched as up-regulated KEGG pathways by CCNY knockdown, whereas neuroactive ligand-receptor interaction, allograft rejection, and autoimmune thyroid disease were significantly enriched as down-regulated KEGG pathways by CCNY overexpression (S3b Fig).

**Fig 4:**
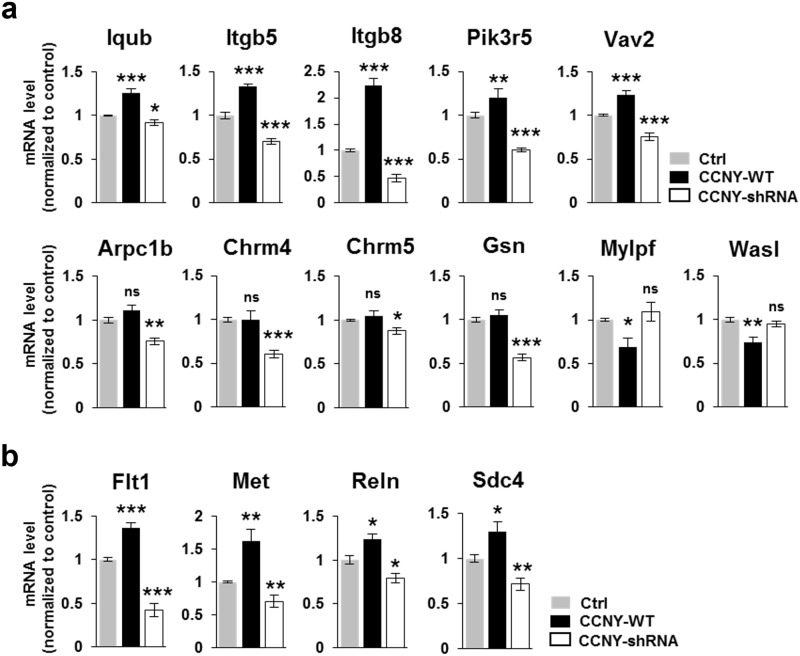
CCNY affects the pathways for the regulation of actin cytoskeleton, focal adhesion, and Extracellular Matrix (ECM)-receptor interaction in hippocampal neurons. (**a**,**b**) Transcript levels of several genes from “regulation of actin cytoskeleton” pathway (**a**) and “focal adhesion” and “ECM-receptor interaction” pathways (**b**) were analyzed by qRT-PCR. n = 3–5 from 3 independent experiments. *p<0.05 relative to control, **p <0.01 relative to control, ***p <0.001 relative to control, ns, not significant, student’s *t* test. Refer the S2 Table.

**Fig 5:**
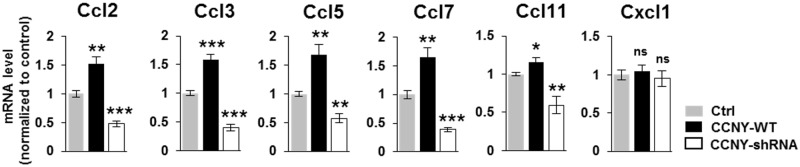
CCNY regulates genes related to Alzheimer’s Disease (AD) in hippocampal neurons. qRT-PCR results of several cytokine genes in the chemokine signaling pathway (S3a Fig) or genes related to AD from CCNY overexpression or knockdown samples. n = 3–5 from 3 independent experiments. *p<0.05 relative to control, **p <0.01 relative to control, ***p <0.001 relative to control, ns, not significant, student’s *t* test. Refer the S2 Table.

Since the role of CCNY in AMPA receptor trafficking and LTP [11] is known to be mediated by actin cytoskeleton [35–38] and CCNY localizes adjacent to the PSD (Fig 1d) [11], we further validated the genes (S2 Table) belonging to the pathways for regulation of actin cytoskeleton (Iqub, Itgb5, Itgb8, Pik3r5, Vav2; Arpc1b, Chrm4, Chrm5, Gsn; Mylpf, Wasl) (Fig 4a; S3, S5 and S6 Figs) and focal adhesion/ECM-receptor interaction (Flt1, Met, Reln, and Sdc4) (Fig 4b; S7 and S8 Figs), respectively, by qRT-PCR. Moreover, previous studies have reported CCNY as an inhibitory regulator for LTP [11], which has been assumed as a cellular model for learning and memory, and the altered levels of several cytokines in memory-deficit Alzheimer’s disease (AD) mouse model [31] that led us to further validate several genes (Ccl2, Ccl7 and Cxcl1; S2 Table) belonging to chemokine signaling pathway in S3a Fig and additional cytokine genes (Ccl3, Ccl5 and Ccl11) reported in the cytokines-AD study [31] (Fig 5). Interestingly, we found that the cytokines that were altered in the AD mouse model [31] were also shown to be regulated by CCNY, suggesting a possible role of CCNY in the AD mouse model.

### Analysis of overlapping DEGs regulated by CCNY overexpression and knockdown

**Fig 6:**
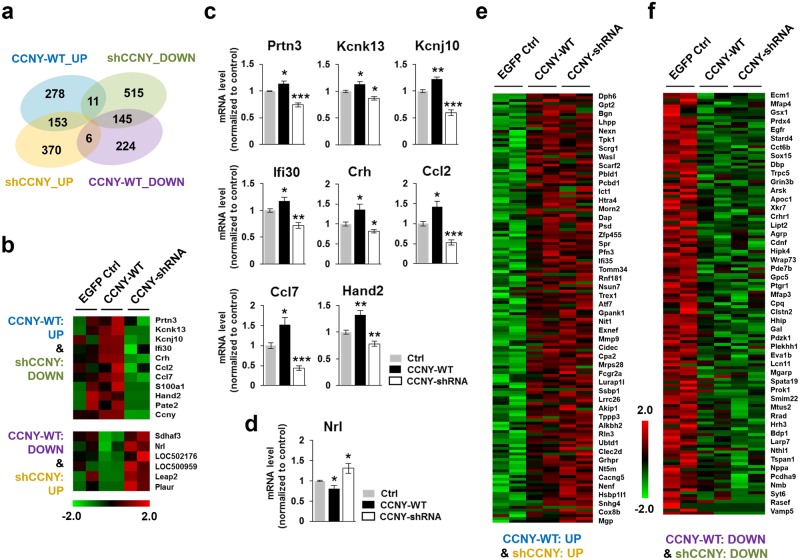
Analysis of overlapping DEGs controlled by CCNY overexpression and knockdown. (**a**) Venn diagram showing the overlaps among the four categories. (**b-d**) Some genes are oppositely regulated by CCNY overexpression and knockdown. (**b**) Heatmap for the genes overlapping up-regulated by CCNY overexpression and down-regulated by CCNY knockdown (upper). Heatmap for the genes overlapping down-regulated by CCNY overexpression and up-regulated by CCNY knockdown (lower). (**c,d**) Several genes from (**b**) were validated by qRT-PCR. n = 3–5 from 3 independent experiments. *p<0.05 relative to control, **p <0.01 relative to control, ***p <0.001 relative to control, ns, not significant, student’s *t* test. (**e**,**f**) Heatmaps for the genes overlapping up-regulated by CCNY overexpression and up-regulated by CCNY knockdown (**e**) and the genes overlapping down-regulated by CCNY overexpression and down-regulated by CCNY knockdown (**f**).

Among 442 DEGs that were up-regulated by CCNY overexpression, 153 and 11 genes were up- and down-regulated, respectively, by CCNY knockdown. Among 375 DEGs that were down-regulated by CCNY overexpression, 145 and 6 genes were down- and up-regulated, respectively, by CCNY knockdown (Fig 6a). mRNA expression levels of several genes (Prtn3, Kcnk13, Kcnj10, Ifi30, Crh, Ccl2, Ccl7 and Hand2) out of 11 that were both up-regulated by CCNY overexpression and down-regulated by CCNY knockdown (Fig 6b and 6c), and a gene (Nrl) out of 6 that were both up-regulated by CCNY knockdown and down-regulated by CCNY overexpression (Fig 6b and 6d) were further validated by qRT-PCR.

The 153 DEGs that were both up-regulated by CCNY overexpression and knockdown (Fig 6e) and the 145 DEGs that were both down-regulated by CCNY overexpression and knockdown (Fig 6f) were further analyzed for GO term enrichment (S4 Fig). It implies that a certain expression level of CCNY is important for physiological maintenance of the GO terms identified in S4 Fig.

## Discussion

In the present study, for the first time, we demonstrated the expression patterns of transcripts targeted by the cyclin protein CCNY in non-proliferating hippocampal neuronal cells. By conducting bioinformatic analysis, including GO terms and KEGG pathways with the RNA-seq data, we examined the CCNY-mediated changes in transcriptome pattern and made subsequent validations on the transcript changes with qRT-PCR in hippocampal neuronal cells. We analyzed total of eight categories of the genes targeted by CCNY overexpression and/or knockdown (Fig 6a): up- or down-regulated by CCNY overexpression, up- or down-regulated by CCNY knockdown, up- or down-regulated by both CCNY overexpression and knockdown, up-regulated by CCNY overexpression and down-regulated by CCNY knockdown, and up-regulated by CCNY knockdown and down-regulated by CCNY overexpression. We thoroughly examined the patterns of analysis results and found that several GO terms and KEGG pathways should be considered significantly valuable for further investigations on their roles particularly in respect to the CCNY-mediated functional phenotypes in neuronal systems.

Through the RNA-seq, transcriptome analysis and qRT-PCR, we presented that actin cytoskeleton, learning or memory, focal adhesion, ECM-receptor interaction, apoptosis, chemokine signaling, and AD-related cytokines are affected by CCNY in neuronal cells. Based on our previous report on the CCNY function in LTP [11] which has been considered as a cellular mechanism for learning and memory and the findings on CCNY regulation of several AD-related cytokines (Fig 5), it is highly plausible that CCNY plays a role in learning and memory-deficit neurological diseases such as AD [39].

The actin cytoskeleton is abundant in the dendritic spines and is an essential factor for spine structure and plasticity [40–44]. In addition, actin dynamics has been reported to play an important role in controlling AMPA receptor trafficking and in bidirectional synaptic plasticity such as LTP and LTD [35–38, 45, 46]. Importantly, CCNY, which was shown to be localized in spines (Fig 1d) [11], has been suggested as an inhibitory regulator for AMPA receptor delivery to synapses during LTP-inducing stimulation [11]. Furthermore, in the present study, the pathway for regulation of actin cytoskeleton was identified to be oppositely regulated by CCNY overexpression and knockdown, and several genes involved in the pathway were qRT-PCR-validated as being regulated by CCNY (S3 Fig and Fig 4a). Therefore, it will be important to provide direct experimental evidence on the role of CCNY in the actin dynamics by investigating the cellular and molecular mechanisms underlying the coordination between CCNY and actin cytoskeleton signaling for neuronal structural and functional plasticity.

The terms and pathways such as DNA replication, mitotic cell cycle, and regulation of cell proliferation were also identified by the DAVID analysis in our experimental systems. Since our culture system used in this study is neurons enriched but glia repressed, those terms and pathways that are related to cell division and are isolated as being targeted by CCNY further suggest and support the idea that cyclin proteins, including CCNY, may play roles in neuronal functions by controlling synapses, synaptic plasticity and/or memory [47] plausibly through the formulation of neuron-specific functional molecular networks.

Transcript expression level of individual genes that we displayed in the present study may indicate the protein expression (mRNA translation) level of the individual genes. Given that CCNY plays a role in synaptic plasticity, the genes isolated as being regulated by CCNY in this study could be involved in the maintenance of synaptic plasticity and memory (re)consolidation, which require new protein synthesis [48–52]. In other words, those genes that did not exhibit any changes by CCNY should not be overlooked since they may be involved in the early stage of synaptic plasticity, which does not require a process of protein synthesis. Therefore, it will be valuable to explore and compare the transcriptome profile changes controlled by CCNY in an activity-dependent manner with several temporal windows after given an activity.

The global and comprehensive bioinformatic analysis of transcriptome controlled by CCNY and consecutive validations with qRT-PCR in neuronal cells revealed some valuable molecules as being regulated by CCNY in neuronal cells and provided useful information on the neuronal and/or synaptic role of CCNY by suggesting several functional pathways. It will require in future to experimentally elucidate the CCNY functions in nervous systems *in vitro* and *in vivo*.

The GO and KEGG pathway analysis adopted in this study provide a meaningful biology associated with a list of genes from a large number of genes through the systematical classifications of DEGs and the statistical over-representation (enrichment) to the functional pathways. However, we cannot overlook the plausibility of other biological meanings that could be missed by the enrichment analysis since the analysis only relies on the known genes and known functions, it cannot reveal unknown functions even in the known genes. Moreover, some biological pathways have been more investigated than others, which relatively builds up more database for the enrichment analysis and thus likely analyzed with more significance than others [27, 28, 53, 54].

## Supporting information

S1 Fig RNA-seq based CCNY expression and Differentially Expressed Genes (DEGs). (**a**) Expression level of CCNY. The values of log_2_FPKM were used. (**b**) The number of DEGs that were up- or down-regulated by CCNY overexpression or knockdown.
(PDF)

S2 Fig Gene Ontology (GO) analysis of CCNY expression level-responsive DEGs. (**a**) GO terms of DEGs up-regulated by CCNY overexpression or down-regulated by CCNY knockdown were analyzed. (**b**) GO terms of DEGs up-regulated by CCNY knockdown or down-regulated by CCNY overexpression were analyzed. *p<0.05, significantly enriched GO terms in DEGs.
(PDF)

S3 Fig KEGG pathway enrichment analysis of CCNY expression level-responsive DEGs. (**a**,**b**) KEGG pathways were analyzed from the DEGs up-regulated by CCNY overexpression or down-regulated by CCNY knockdown (**a**) and the DEGs up-regulated by CCNY knockdown or down-regulated by CCNY overexpression (**b**). *p<0.05, significantly enriched KEGG pathways in DEGs. The Y-axes indicate the pathway categories, and the X-axes indicate the enrichment of the pathways.
(PDF)

S4 Fig GO term enrichment analysis of DEGs in Fig 6e and 6f. GO analysis for the 153 DEGs that were both up-regulated by CCNY overexpression and knockdown and for the 145 DEGs that were both down-regulated by CCNY overexpression and knockdown. *p<0.05, significantly enriched GO terms in DEGs.
(PDF)

S5 Fig KEGG pathways of regulation of actin cytoskeleton. Red stars mark up-regulated mRNAs targeted by CCNY overexpression in cultured hippocampal neurons.
(PDF)

S6 Fig KEGG pathways of regulation of actin cytoskeleton. Red stars mark down-regulated mRNAs targeted by CCNY knockdown in cultured hippocampal neurons.
(PDF)

S7 Fig KEGG pathways of focal adhesion. Red stars mark down-regulated mRNAs targeted by CCNY knockdown in cultured hippocampal neurons.
(PDF)

S8 Fig KEGG pathways of ECM-receptor interaction. Red stars mark down-regulated mRNAs targeted by CCNY knockdown in cultured hippocampal neurons.
(PDF)

S9 Fig Original blots for immunoblot analysis in Fig 1c. (PDF)

S1 Table Examples of genes that belong to GO terms in S2 Fig. (PDF)

S2 Table Examples of genes that belong to KEGG pathways in S3 Fig. (PDF)

S3 Table Oligonucleotides used for quantitative real-time PCR. (PDF)

## References

[1] Evans T, Rosenthal ET, Youngblom J, Distel D, Hunt T. Cyclin: a protein specified by maternal mRNA in sea urchin eggs that is destroyed at each cleavage division. Cell. 1983;33(2):389–96. 6134587 10.1016/0092-8674(83)90420-8

[2] Liu D, Guest S, Finley RL Jr. Why cyclin Y? A highly conserved cyclin with essential functions. Fly (Austin). 2010;4(4):278–82. 20699655 10.4161/fly.4.4.12881 PMC3174478

[3] Noble ME, Endicott JA, Brown NR, Johnson LN. The cyclin box fold: protein recognition in cell-cycle and transcription control. Trends Biochem Sci. 1997;22(12):482–7. 9433129 10.1016/s0968-0004(97)01144-4

[4] Jiang M, Gao Y, Yang T, Zhu X, Chen J. Cyclin Y, a novel membrane-associated cyclin, interacts with PFTK1. FEBS Lett. 2009;583(13):2171–8. 10.1016/j.febslet.2009.06.010 19524571

[5] Xu Y, Wang Z, Wang J, Li J, Wang H, Yue W. Lentivirus-mediated knockdown of cyclin Y (CCNY) inhibits glioma cell proliferation. Oncol Res. 2010;18(8):359–64. 20441050 10.3727/096504010x12644422320582

[6] Yue W, Zhao X, Zhang L, Xu S, Liu Z, Ma L, et al Cell cycle protein cyclin Y is associated with human non-small-cell lung cancer proliferation and tumorigenesis. Clin Lung Cancer. 2011;12(1):43–50. 10.3816/CLC.2011.n.006 21273179

[7] Zeng L, Cai C, Li S, Wang W, Li Y, Chen J, et al Essential Roles of Cyclin Y-Like 1 and Cyclin Y in Dividing Wnt-Responsive Mammary Stem/Progenitor Cells. PLoS Genet. 2016;12(5):e1006055 10.1371/journal.pgen.1006055 27203244 PMC4874687

[8] An W, Zhang Z, Zeng L, Yang Y, Zhu X, Wu J. Cyclin Y Is Involved in the Regulation of Adipogenesis and Lipid Production. PLoS One. 2015;10(7):e0132721 10.1371/journal.pone.0132721 26161966 PMC4498623

[9] Ou CY, Poon VY, Maeder CI, Watanabe S, Lehrman EK, Fu AK, et al Two cyclin-dependent kinase pathways are essential for polarized trafficking of presynaptic components. Cell. 2010;141(5):846–58. 10.1016/j.cell.2010.04.011 20510931 PMC3168554

[10] Park M, Watanabe S, Poon VY, Ou CY, Jorgensen EM, Shen K. CYY-1/cyclin Y and CDK-5 differentially regulate synapse elimination and formation for rewiring neural circuits. Neuron. 2011;70(4):742–57. 10.1016/j.neuron.2011.04.002 21609829 PMC3168547

[11] Cho E, Kim D-H, Hur Y-N, Whitcomb DJ, Regan P, Hong J-H, et al Cyclin Y inhibits plasticity-induced AMPA receptor exocytosis and LTP. Sci Rep. 2015;5. 10.1038/srep12624 PMC4518236 26220330

[12] Bliss TV, Lomo T. Long-lasting potentiation of synaptic transmission in the dentate area of the anaesthetized rabbit following stimulation of the perforant path. J Physiol. 1973;232(2):331–56. 4727084 10.1113/jphysiol.1973.sp010273 PMC1350458

[13] Luscher C, Xia H, Beattie EC, Carroll RC, von Zastrow M, Malenka RC, et al Role of AMPA receptor cycling in synaptic transmission and plasticity. Neuron. 1999;24(3):649–58. 10595516 10.1016/s0896-6273(00)81119-8

[14] Maletic-Savatic M, Malinow R, Svoboda K. Rapid dendritic morphogenesis in CA1 hippocampal dendrites induced by synaptic activity. Science. 1999;283(5409):1923–7. 10082466 10.1126/science.283.5409.1923

[15] Matsuzaki M, Honkura N, Ellis-Davies GC, Kasai H. Structural basis of long-term potentiation in single dendritic spines. Nature. 2004;429(6993):761–6. 10.1038/nature02617 15190253 PMC4158816

[16] Nagalakshmi U, Wang Z, Waern K, Shou C, Raha D, Gerstein M, et al The transcriptional landscape of the yeast genome defined by RNA sequencing. Science. 2008;320(5881):1344–9. 10.1126/science.1158441 18451266 PMC2951732

[17] Shen GM, Dou W, Niu JZ, Jiang HB, Yang WJ, Jia FX, et al Transcriptome analysis of the oriental fruit fly (Bactrocera dorsalis). PLoS One. 2011;6(12):e29127 10.1371/journal.pone.0029127 22195006 PMC3240649

[18] Sultan M, Schulz MH, Richard H, Magen A, Klingenhoff A, Scherf M, et al A global view of gene activity and alternative splicing by deep sequencing of the human transcriptome. Science. 2008;321(5891):956–60. 10.1126/science.1160342 18599741

[19] Chen BJ, Mills JD, Janitz C, Janitz M. RNA-Sequencing to Elucidate Early Patterns of Dysregulation Underlying the Onset of Alzheimer's Disease. Methods Mol Biol. 2016;1303:327–47. 10.1007/978-1-4939-2627-5_20 26235077

[20] Courtney E, Kornfeld S, Janitz K, Janitz M. Transcriptome profiling in neurodegenerative disease. J Neurosci Methods. 2010;193(2):189–202. 10.1016/j.jneumeth.2010.08.018 20800617

[21] Lin L, Park JW, Ramachandran S, Zhang Y, Tseng YT, Shen S, et al Transcriptome sequencing reveals aberrant alternative splicing in Huntington's disease. Hum Mol Genet. 2016. 10.1093/hmg/ddw187 PMC5179942 27378699

[22] Miller JR, Lo KK, Andre R, Hensman Moss DJ, Trager U, Stone TC, et al RNA-Seq of Huntington's disease patient myeloid cells reveals innate transcriptional dysregulation associated with proinflammatory pathway activation. Hum Mol Genet. 2016. 10.1093/hmg/ddw142 PMC5181590 27170315

[23] Baek SJ, Kim M, Bae DH, Kim JH, Kim HJ, Han ME, et al Integrated epigenomic analyses of enhancer as well as promoter regions in gastric cancer. Oncotarget. 2016. 10.18632/oncotarget.8239 PMC5041931 27016420

[24] Kim D, Pertea G, Trapnell C, Pimentel H, Kelley R, Salzberg SL. TopHat2: accurate alignment of transcriptomes in the presence of insertions, deletions and gene fusions. Genome Biol. 2013;14(4):R36 10.1186/gb-2013-14-4-r36 23618408 PMC4053844

[25] Trapnell C, Williams BA, Pertea G, Mortazavi A, Kwan G, van Baren MJ, et al Transcript assembly and quantification by RNA-Seq reveals unannotated transcripts and isoform switching during cell differentiation. Nat Biotechnol. 2010;28(5):511–5. 10.1038/nbt.1621 20436464 PMC3146043

[26] Howe EA, Sinha R, Schlauch D, Quackenbush J. RNA-Seq analysis in MeV. Bioinformatics. 2011;27(22):3209–10. 10.1093/bioinformatics/btr490 21976420 PMC3208390

[27] Huang da W, Sherman BT, Lempicki RA. Systematic and integrative analysis of large gene lists using DAVID bioinformatics resources. Nat Protoc. 2009;4(1):44–57. 10.1038/nprot.2008.211 19131956

[28] Huang da W, Sherman BT, Lempicki RA. Bioinformatics enrichment tools: paths toward the comprehensive functional analysis of large gene lists. Nucleic Acids Res. 2009;37(1):1–13. 10.1093/nar/gkn923 19033363 PMC2615629

[29] Kanehisa M, Goto S. KEGG: kyoto encyclopedia of genes and genomes. Nucleic Acids Res. 2000;28(1):27–30. 10592173 10.1093/nar/28.1.27 PMC102409

[30] Kanehisa M, Goto S, Sato Y, Kawashima M, Furumichi M, Tanabe M. Data, information, knowledge and principle: back to metabolism in KEGG. Nucleic Acids Res. 2014;42(Database issue):D199–205. 10.1093/nar/gkt1076 24214961 PMC3965122

[31] Yang SH, Kim J, Lee MJ, Kim Y. Abnormalities of plasma cytokines and spleen in senile APP/PS1/Tau transgenic mouse model. Sci Rep. 2015;5:15703 10.1038/srep15703 26503550 PMC4621607

[32] Ashburner M, Ball CA, Blake JA, Botstein D, Butler H, Cherry JM, et al Gene ontology: tool for the unification of biology. The Gene Ontology Consortium. Nat Genet. 2000;25(1):25–9. 10.1038/75556 10802651 PMC3037419

[33] Jo J, Whitcomb DJ, Olsen KM, Kerrigan TL, Lo SC, Bru-Mercier G, et al Abeta(1–42) inhibition of LTP is mediated by a signaling pathway involving caspase-3, Akt1 and GSK-3beta. Nat Neurosci. 2011;14(5):545–7. 10.1038/nn.2785 21441921

[34] Li Z, Jo J, Jia JM, Lo SC, Whitcomb DJ, Jiao S, et al Caspase-3 activation via mitochondria is required for long-term depression and AMPA receptor internalization. Cell. 2010;141(5):859–71. 10.1016/j.cell.2010.03.053 20510932 PMC2909748

[35] Bosch M, Castro J, Saneyoshi T, Matsuno H, Sur M, Hayashi Y. Structural and molecular remodeling of dendritic spine substructures during long-term potentiation. Neuron. 2014;82(2):444–59. 10.1016/j.neuron.2014.03.021 24742465 PMC4281348

[36] Bramham CR. Local protein synthesis, actin dynamics, and LTP consolidation. Curr Opin Neurobiol. 2008;18(5):524–31. 10.1016/j.conb.2008.09.013 18834940

[37] Hanley JG. Actin-dependent mechanisms in AMPA receptor trafficking. Front Cell Neurosci. 2014;8:381 10.3389/fncel.2014.00381 25429259 PMC4228833

[38] Okamoto K, Nagai T, Miyawaki A, Hayashi Y. Rapid and persistent modulation of actin dynamics regulates postsynaptic reorganization underlying bidirectional plasticity. Nat Neurosci. 2004;7(10):1104–12. 10.1038/nn1311 15361876

[39] Cho E, Park M. Palmitoylation in Alzheimer's disease and other neurodegenerative diseases. Pharmacol Res. 2016;111:133–51. 10.1016/j.phrs.2016.06.008 27293050

[40] Blanpied TA, Kerr JM, Ehlers MD. Structural plasticity with preserved topology in the postsynaptic protein network. Proc Natl Acad Sci U S A. 2008;105(34):12587–92. 10.1073/pnas.0711669105 18723686 PMC2519044

[41] Cingolani LA, Goda Y. Actin in action: the interplay between the actin cytoskeleton and synaptic efficacy. Nat Rev Neurosci. 2008;9(5):344–56. 10.1038/nrn2373 18425089

[42] Hotulainen P, Hoogenraad CC. Actin in dendritic spines: connecting dynamics to function. J Cell Biol. 2010;189(4):619–29. 10.1083/jcb.201003008 20457765 PMC2872912

[43] Lise MF, Wong TP, Trinh A, Hines RM, Liu L, Kang R, et al Involvement of myosin Vb in glutamate receptor trafficking. J Biol Chem. 2006;281(6):3669–78. 10.1074/jbc.M511725200 16338934

[44] Wolf M, Zimmermann AM, Gorlich A, Gurniak CB, Sassoe-Pognetto M, Friauf E, et al ADF/Cofilin Controls Synaptic Actin Dynamics and Regulates Synaptic Vesicle Mobilization and Exocytosis. Cereb Cortex. 2015;25(9):2863–75. 10.1093/cercor/bhu081 24770705

[45] Kerr JM, Blanpied TA. Subsynaptic AMPA receptor distribution is acutely regulated by actin-driven reorganization of the postsynaptic density. J Neurosci. 2012;32(2):658–73. 10.1523/JNEUROSCI.2927-11.2012 22238102 PMC3596168

[46] Wang Z, Edwards JG, Riley N, Provance DW Jr., Karcher R, Li XD, et al Myosin Vb mobilizes recycling endosomes and AMPA receptors for postsynaptic plasticity. Cell. 2008;135(3):535–48. 10.1016/j.cell.2008.09.057 18984164 PMC2585749

[47] Odajima J, Wills ZP, Ndassa YM, Terunuma M, Kretschmannova K, Deeb TZ, et al Cyclin E constrains Cdk5 activity to regulate synaptic plasticity and memory formation. Dev Cell. 2011;21(4):655–68. 10.1016/j.devcel.2011.08.009 21944720 PMC3199337

[48] Bonin RP, De Koninck Y. Reconsolidation and the regulation of plasticity: moving beyond memory. Trends Neurosci. 2015;38(6):336–44. 10.1016/j.tins.2015.04.007 25987442

[49] Clopath C. Synaptic consolidation: an approach to long-term learning. Cogn Neurodyn. 2012;6(3):251–7. 10.1007/s11571-011-9177-6 23730356 PMC3368062

[50] Nguyen PV, Abel T, Kandel ER. Requirement of a critical period of transcription for induction of a late phase of LTP. Science. 1994;265(5175):1104–7. 8066450 10.1126/science.8066450

[51] Rosenberg T, Gal-Ben-Ari S, Dieterich DC, Kreutz MR, Ziv NE, Gundelfinger ED, et al The roles of protein expression in synaptic plasticity and memory consolidation. Front Mol Neurosci. 2014;7:86 10.3389/fnmol.2014.00086 25429258 PMC4228929

[52] Rosenblum K, Meiri N, Dudai Y. Taste memory: the role of protein synthesis in gustatory cortex. Behav Neural Biol. 1993;59(1):49–56. 8442732 10.1016/0163-1047(93)91145-d

[53] Khatri P, Draghici S. Ontological analysis of gene expression data: current tools, limitations, and open problems. Bioinformatics. 2005;21(18):3587–95. 10.1093/bioinformatics/bti565 15994189 PMC2435250

[54] Tarca AL, Bhatti G, Romero R. A comparison of gene set analysis methods in terms of sensitivity, prioritization and specificity. PLoS One. 2013;8(11):e79217 10.1371/journal.pone.0079217 24260172 PMC3829842

