# Supplementary material for: Cyclin Y-mediated transcript profiling reveals several important functional pathways regulated by Cyclin Y in hippocampal neurons
Source: PLoS One. 2017 Feb 27;12(2):e0172547. doi: 10.1371/journal.pone.0172547 (PMC5328252; doi:10.1371/journal.pone.0172547)
Supplement: S1 Fig — (a) Expression level of CCNY. The values of log2FPKM were used. (b) The number of DEGs that were up- or down-regulated by CCNY overexpression or knockdown. (PDF) [file pone.0172547.s001.pdf]

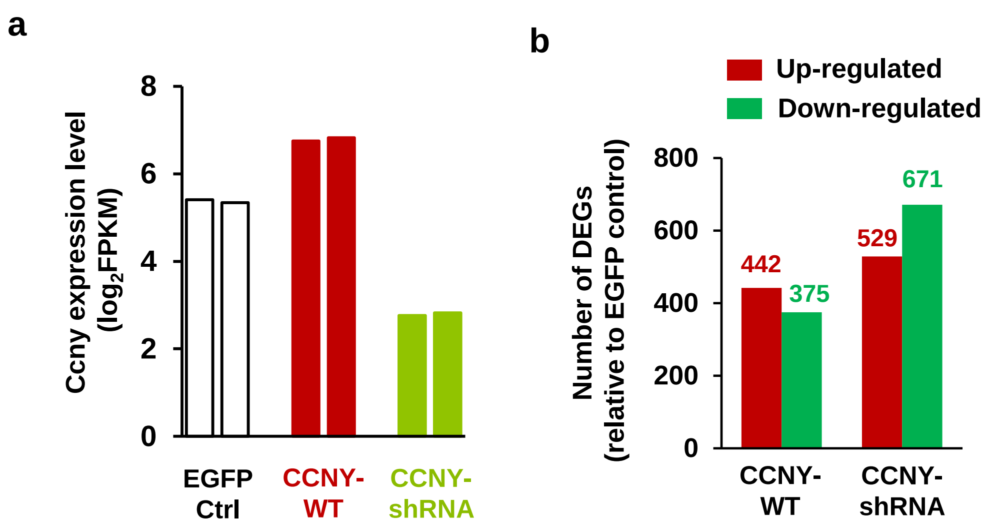

S1 Fig. RNA-seq based CCNY expression and differentially expressed genes (DEGs). (**a**) Expression level of CCNY. The values of log_2FPKM were used. (**b**) The number of DEGs that were up- or down-regulated by CCNY overexpression or knockdown.
