# Supplementary material for: Cyclin Y-mediated transcript profiling reveals several important functional pathways regulated by Cyclin Y in hippocampal neurons
Source: PLoS One. 2017 Feb 27;12(2):e0172547. doi: 10.1371/journal.pone.0172547 (PMC5328252; doi:10.1371/journal.pone.0172547)
Supplement: S2 Fig — (a) GO terms of DEGs up-regulated by CCNY overexpression or down-regulated by CCNY knockdown were analyzed. (b) GO terms of DEGs up-regulated by CCNY knockdown or down-regulated by CCNY overexpression were analyzed. *p<0.05, significantly enriched GO terms in DEGs. (PDF) [file pone.0172547.s002.pdf]

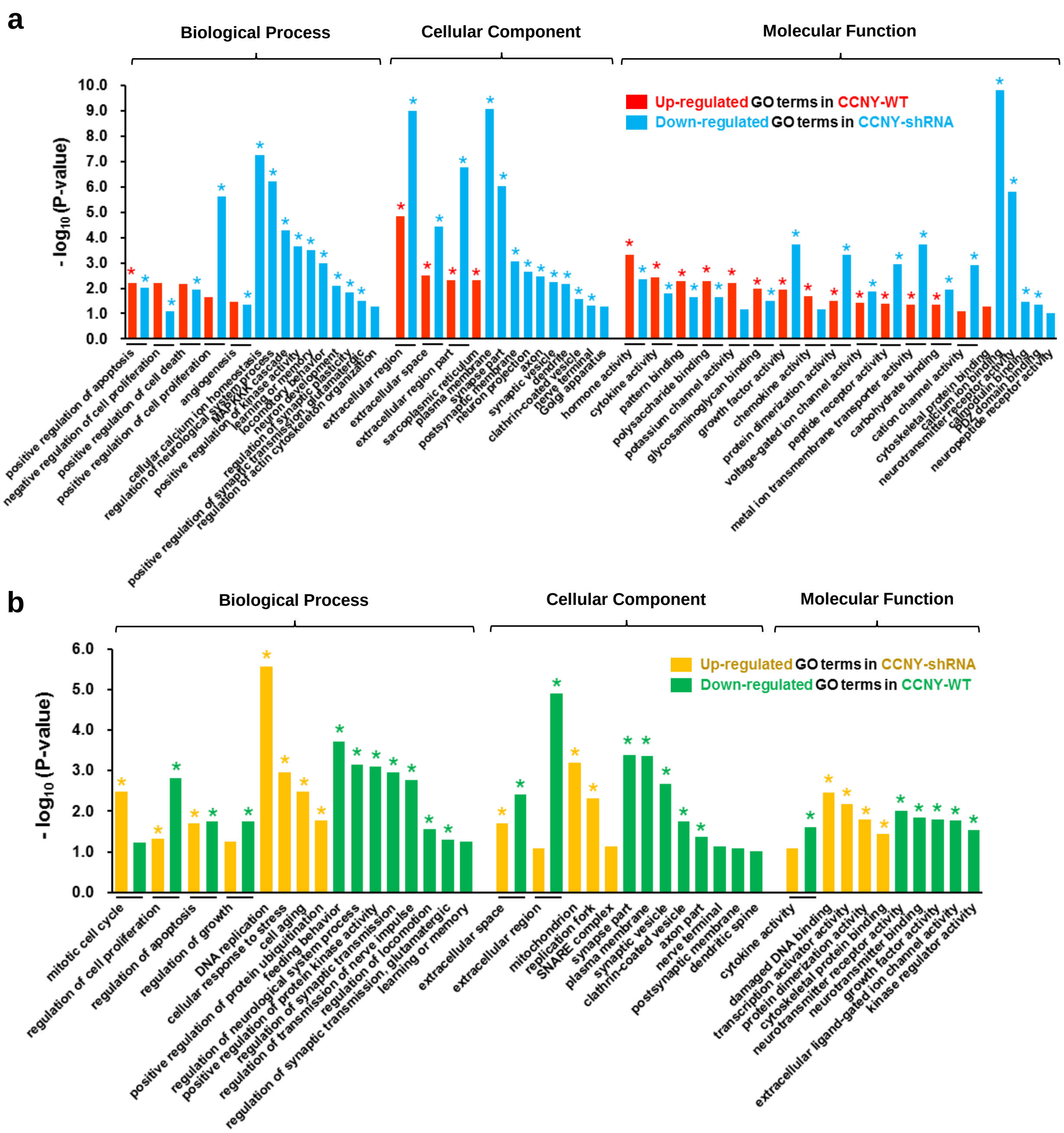

S2 Fig. Gene ontology (GO) analysis of CCNY expression level–responsive DEGs. (**a**) GO terms of DEGs up-regulated by CCNY overexpression or down-regulated by CCNY knockdown were analyzed. (**b**) GO terms of DEGs up-regulated by CCNY knockdown or down-regulated by CCNY overexpression were analyzed. *p<0.05, significantly enriched GO terms in DEGs.
