# Supplementary material for: Cyclin Y-mediated transcript profiling reveals several important functional pathways regulated by Cyclin Y in hippocampal neurons
Source: PLoS One. 2017 Feb 27;12(2):e0172547. doi: 10.1371/journal.pone.0172547 (PMC5328252; doi:10.1371/journal.pone.0172547)
Supplement: S3 Fig — (a,b) KEGG pathways were analyzed from the DEGs up-regulated by CCNY overexpression or down-regulated by CCNY knockdown (a) and the DEGs up-regulated by CCNY knockdown or down-regulated by CCNY overexpression (b). *p<0.05, significantly enriched KEGG pathways in DEGs. The Y-axes indicate the pathway categories, and the X-axes indicate the enrichment of the pathways. (PDF) [file pone.0172547.s003.pdf]

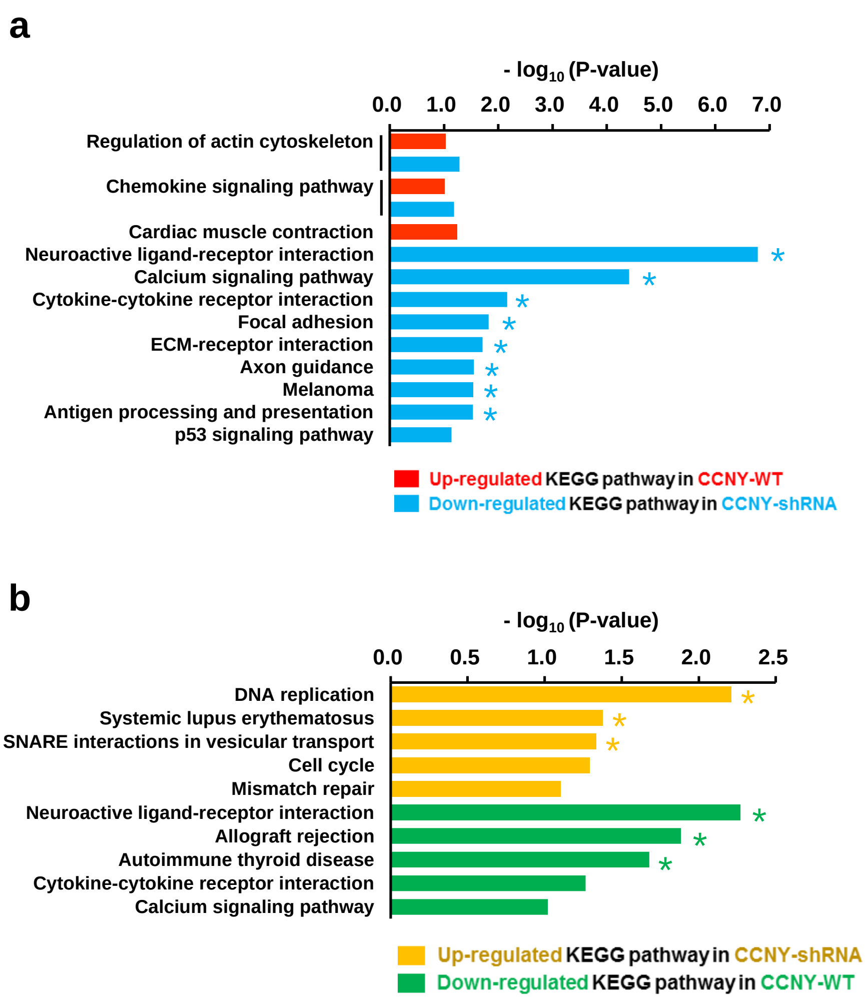

S3 Fig. KEGG pathway enrichment analysis of CCNY expression level–responsive DEGs. (**a**,**b**) KEGG pathways were analyzed from the DEGs up-regulated by CCNY overexpression or down-regulated by CCNY knockdown (**a**) and the DEGs up-regulated by CCNY knockdown or down-regulated by CCNY overexpression (**b**). *p<0.05, significantly enriched KEGG pathways in DEGs. The Y-axes indicate the pathway categories, and the X-axes indicate the enrichment of the pathways.
