# Supplementary material for: Cyclin Y-mediated transcript profiling reveals several important functional pathways regulated by Cyclin Y in hippocampal neurons
Source: PLoS One. 2017 Feb 27;12(2):e0172547. doi: 10.1371/journal.pone.0172547 (PMC5328252; doi:10.1371/journal.pone.0172547)
Supplement: S4 Fig — GO analysis for the 153 DEGs that were both up-regulated by CCNY overexpression and knockdown and for the 145 DEGs that were both down-regulated by CCNY overexpression and knockdown. *p<0.05, significantly enriched GO terms in DEGs. (PDF) [file pone.0172547.s004.pdf]

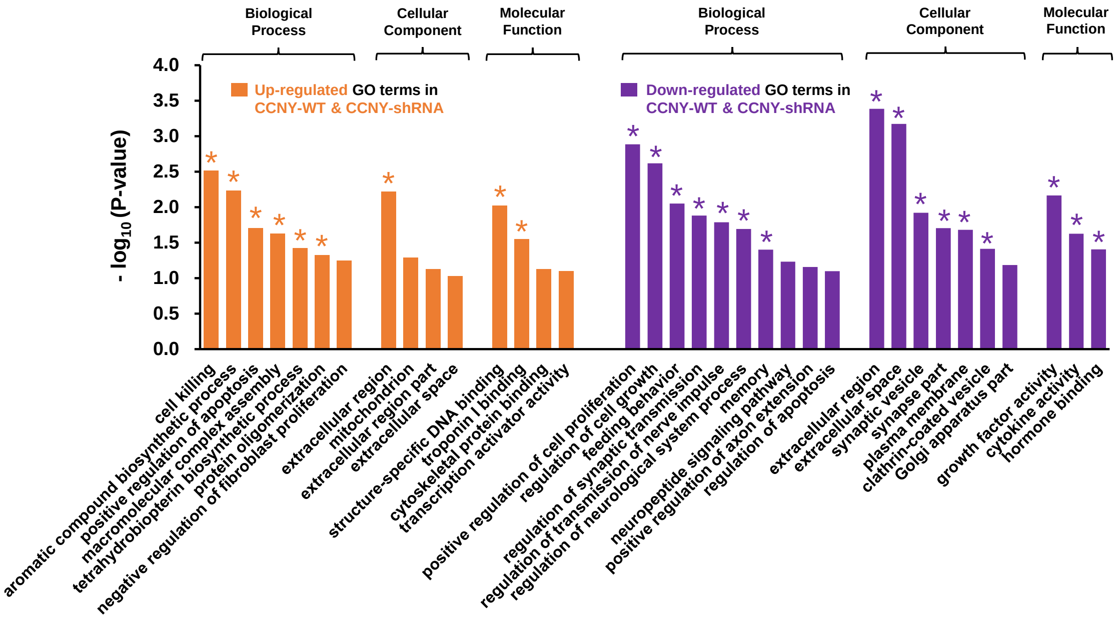

S4 Fig. GO term enrichment analysis of DEGs in Figs 6e and 6f. GO analysis for the 153 DEGs that were both up-regulated by CCNY overexpression and knockdown and for the 145 DEGs that were both down-regulated by CCNY overexpression and knockdown. *$p<0.05$, significantly enriched GO terms in DEGs.
