# Supplementary material for: Cyclin Y-mediated transcript profiling reveals several important functional pathways regulated by Cyclin Y in hippocampal neurons
Source: PLoS One. 2017 Feb 27;12(2):e0172547. doi: 10.1371/journal.pone.0172547 (PMC5328252; doi:10.1371/journal.pone.0172547)
Supplement: S5 Fig — Red stars mark up-regulated mRNAs targeted by CCNY overexpression in cultured hippocampal neurons. (PDF) [file pone.0172547.s005.pdf]

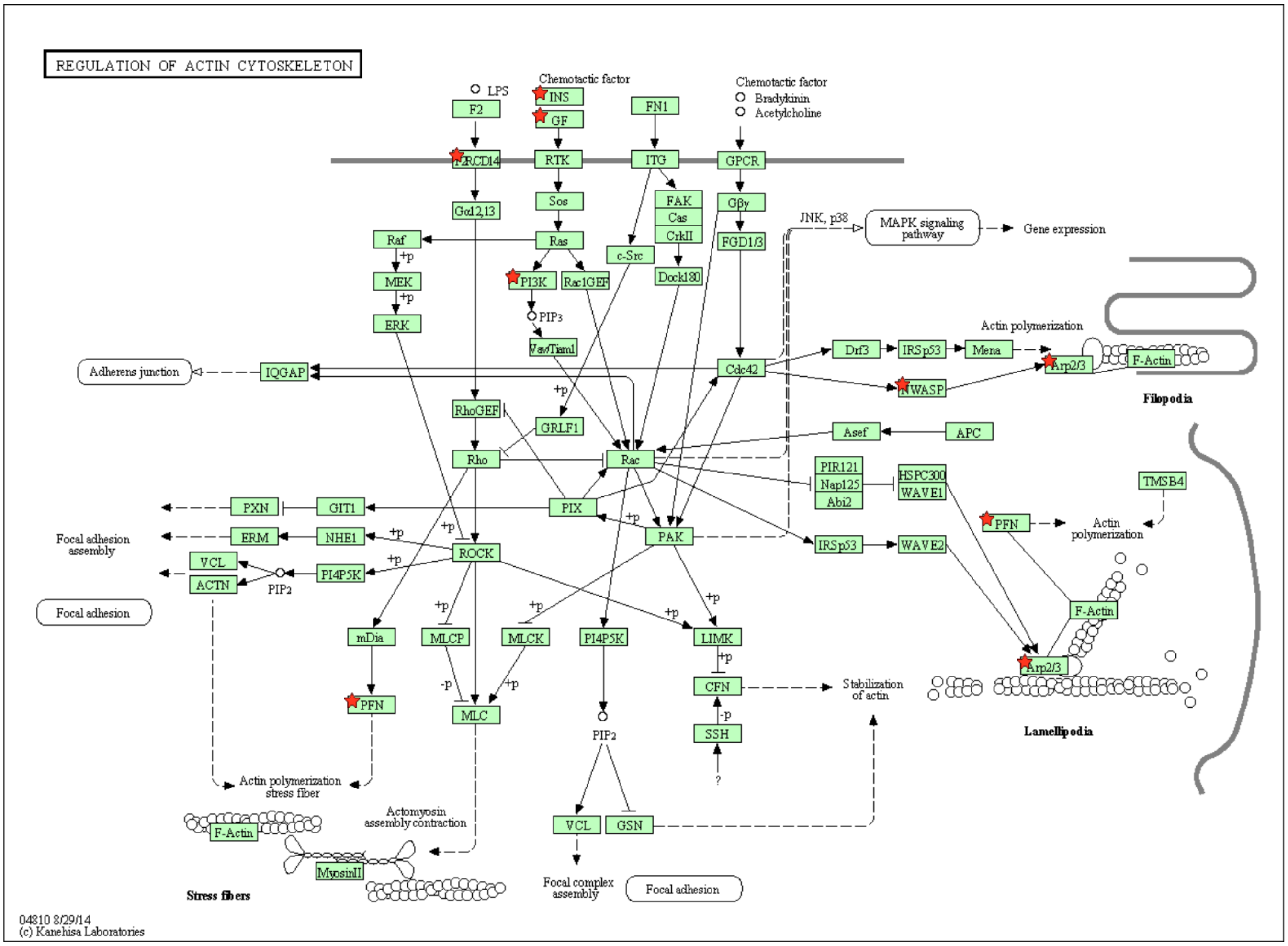


**S5 Fig. KEGG pathways of regulation of actin cytoskeleton.** Red stars mark up-regulated mRNAs targeted by CCNY overexpression in cultured hippocampal neurons.
