# Supplementary figures and images for: Cyclin Y-mediated transcript profiling reveals several important functional pathways regulated by Cyclin Y in hippocampal neurons
Source: PLoS One. 2017 Feb 27;12(2):e0172547. doi: 10.1371/journal.pone.0172547 (PMC5328252; doi:10.1371/journal.pone.0172547)

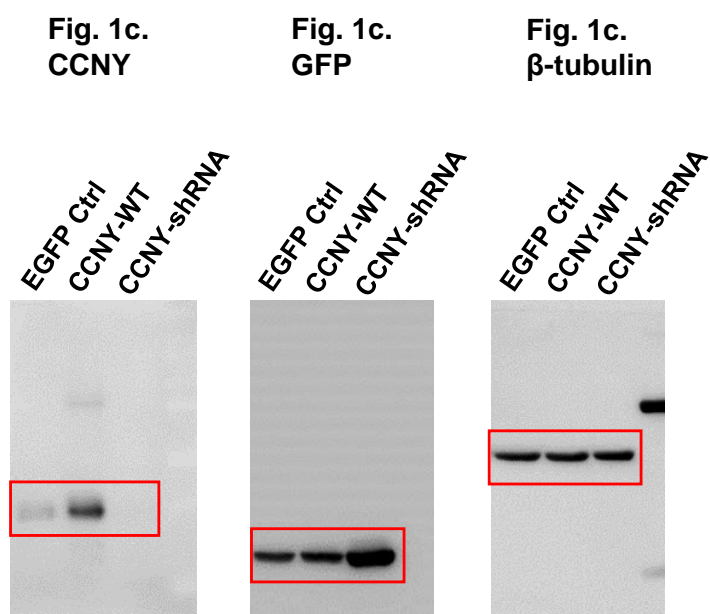

**S9 Fig. Original blots for immunoblot analysis in Fig 1c.**

Supplement: S9 Fig — (PDF) [file pone.0172547.s009.pdf]
