# Supplementary material for: Cyclin Y-mediated transcript profiling reveals several important functional pathways regulated by Cyclin Y in hippocampal neurons
Source: PLoS One. 2017 Feb 27;12(2):e0172547. doi: 10.1371/journal.pone.0172547 (PMC5328252; doi:10.1371/journal.pone.0172547)
Supplement: S1 Table — (PDF) [file pone.0172547.s010.pdf]

S1 Table. Examples of genes that belong to GO terms in S2 Fig.

GO term	Category	Genes	P-value
positive regulation of apoptosis	Up-regulated GO terms in CCNY-WT	**<u>Mmp9</u>**, Cebpg, Lgals7, Tp63, Inha, Pmaip1, Cidec, **<u>Nupr1</u>**, Il12a, Pycard, **<u>Crh</u>**, Ubb, Ngfr, **<u>Fcgr2a</u>**, Id3	0.0062
	Down-regulated GO terms in CCNY-shRNA	Prkca, Fgfr3, Ptgs2, Grin2a, Nr4a1, **<u>Chek2</u>**, Stat1, Gal, Timp3, Tradd, **<u>Gch1</u>**, Xpa, Cd44, Psen1, Lck, **<u>Crh</u>**, Sort1, Cd24, Tp53inp1, Ngf	0.0096
regulation of apoptosis	Down-regulated GO terms in CCNY-WT	Il4, B4galt1, Egfr, Gfral, Phb, **<u>Sphk1</u>**, **<u>Rxfp2</u>**, Nr4a1, Gal, Prkcd, Tnfrsf4, **<u>Crhr1</u>**, P2rx4, Endog, Ern1, Ghrl, Rab26, Spn, Ngf	0.0183
	Up-regulated GO terms in CCNY-shRNA	Txnip, Htatip2, **<u>Gnrh1</u>**, **<u>Mmp9</u>**, Cebpg, Aph1b, **<u>Btc</u>**, Cideb, Esr1, Tp63, Cidea, Fadd, Pmaip1, Sod1, Gdnf, Bcl2l11, Mif, Cidec, **<u>Acvr1c</u>**, Ednrb, Myd88, **<u>Nupr1</u>**, **<u>Fcgr2a</u>**, Dcun1d3	0.0195
learning or memory	Down-regulated GO terms in CCNY-WT	**<u>Crhr1</u>**, Egr1, **<u>Hrh3</u>**, **<u>Drd2</u>**, Ghrl, Ngf	0.0550
	Down-regulated GO terms in CCNY-shRNA	Prkca, **<u>Klk8</u>**, **<u>Slc6a1</u>**, Ptgs2, Grin2a, **<u>Pcdh8</u>**, Ephb2, **<u>Crhr1</u>**, Grm4, Psen1, **<u>Hrh3</u>**, **<u>Bche</u>**, **<u>Crh</u>**, Ngf	0.0003
regulation of synaptic plasticity	Down-regulated GO terms in CCNY-shRNA	**<u>Crhr1</u>**, Atp2b2, Adrb1, Ptgs2, Psen1, Grin2a, Bcan, Ephb2	0.0147

*Note that qRT-PCR validated genes are bold underlined.
