# Supplementary material for: Cyclin Y-mediated transcript profiling reveals several important functional pathways regulated by Cyclin Y in hippocampal neurons
Source: PLoS One. 2017 Feb 27;12(2):e0172547. doi: 10.1371/journal.pone.0172547 (PMC5328252; doi:10.1371/journal.pone.0172547)
Supplement: S2 Table — (PDF) [file pone.0172547.s011.pdf]

S2 Table. Examples of genes that belong to KEGG pathways in S3 Fig.

KEGG pathway	Category	Genes	P-value
Regulation of actin cytoskeleton	Up-regulated KEGG pathways in CCNY-WT	**Arpc1b**, Pfn3, Fgf23, **Pik3r5**, **Wasl**, Ins2, Fgf1, F2r	0.0934
	Down-regulated KEGG pathways in CCNY-shRNA	Egfr, **Chrm5**, Fgfr3, **Chrm4**, **Gsn**, **Itgb8**, Fgf16, **Itgb5**, **Mylpf**, Fgf22, Pdgfc, **Vav2**, **Iqub**	0.0515
Chemokine signaling pathway	Up-regulated KEGG pathways in CCNY-WT	Ccl1, **Cxcl1**, **Ccl2**, **Pik3r5**, **Wasl**, **Ccl7**, Cxcl10	0.0982
	Down-regulated KEGG pathways in CCNY-shRNA	**Ccl2**, Prex1, Ccr10, Rasgrp2, **Vav2**, Stat1, Cxcl12, Prkx, **Ccl7**, **Iqub**, Shc4	0.0651
Cytokine-cytokine receptor interaction	Down-regulated KEGG pathways in CCNY-shRNA	Il4, Egfr, **Flt1**, **Ccl2**, **Met**, Hgf, Cxcl12, Lif, Acvr2a, Ccr10, Eda, Ltb, Ifngr2, Ifngr1, Bmpr1a	0.0068
Focal adhesion	Down-regulated KEGG pathways in CCNY-shRNA	Prkca, Egfr, **Flt1**, **Met**, **Itgb5**, **Mylpf**, Hgf, **Vav2**, **Itgb8**, **Reln**, Pdgfc, Col11a2, Col11a1, Shc4	0.0150
ECM-receptor interaction	Down-regulated KEGG pathways in CCNY-shRNA	Cd44, **Itgb8**, **Itgb5**, **Reln**, Col11a2, **Sdc4**, Col11a1, Sv2c	0.0196

*Note that qRT-PCR validated genes are bold underlined.
