# Supplementary material for: Cyclin Y-mediated transcript profiling reveals several important functional pathways regulated by Cyclin Y in hippocampal neurons
Source: PLoS One. 2017 Feb 27;12(2):e0172547. doi: 10.1371/journal.pone.0172547 (PMC5328252; doi:10.1371/journal.pone.0172547)
Supplement: S3 Table — (PDF) [file pone.0172547.s012.pdf]

S3 Table. Oligonucleotides used for quantitative real-time PCR.

Gene	Forward primer (5' to 3')	Reverse primer (5' to 3')	*Acc. No.
Acvr1c	TCTCCAGATGCCCCTAGACT	CTTCCTGTATGTGCACTGGC	NM_139090
Arpc1b	TGGCCTCTGAGACATTACCG	CACCAAAGCTCAATGTCCCC	NM_019289
Bche	ATATGCCCTGCACTGGAGTT	GCCATGCATTACTCCCATCC	NM_022942
Btc	GGACGAACAAACTCCCTCCT	CCATGACGCCTATCAAGCAG	NM_022256
Ccl11	TTCCATCCCAACTTCCTGCT	TATGGCTTTCAGCGTGCATC	NM_019205
Ccl2	AGTTAATGCCCCACTCACCT	CAGCTTCTTTGGGACACCTG	NM_031530
Ccl3	CTGCCTGCTGCTTCTCCTAT	GATCTGCCGGTTTCTCTTGG	NM_013025
Ccl5	ATATGGCTCGGACACCACTC	CGAGTGACAAAGACGACTGC	NM_031116
Ccl7	CCAACCAGATGGGACCAATTC	ACTTCCATGCCCTTTTTGGTC	NM_001007612
Ccny	GCCTCTAAGGTGTGGGATGA	GCAATTCAAGGAACTGGCGT	NM_001191833
Chek2	ACAACTACTGGTTCGGGAGG	TAGGGCCCATTTCCCTGAAG	NM_053677
Chrm4	ACGTCAACAGCACCATCAAC	TGTGCCGATGTTCCGATACT	NM_031547
Chrm5	ACCCCATCTGCTATGCTCTC	AGGGTAGCTTGCTGTTTCCT	NM_017362
Crh	ATTTCTTGCAACCGGAGCAG	AGCGGGACTTCTGTTGAGG	NM_031019
Crhr1	CCGCTACAACACGACAAACA	CTTCGTTGAGAATCTCCTGGC	NM_112261
Cxcl1	CAGACAGTGGCAGGGATTCA	ATCTTCTGAACCATGGGGGC	NM_030845
Drd2	ACAGGCCCCACTACAACTAC	GTCTGCAAAGCCTTCTCTCG	NM_012547
Fcgr2a	AGGTGCCATAGCTGGAAGAA	TGTGACTGTGGTTGGCTTTG	NM_001135992
Flt1	TGCAGGAAACCATAGCAGGA	AGATTTCTCCGTTGCGGGTA	NM_019306
Gapdh	ACATCATCCCTGCATCCACT	ACAACGGATACATTGGGGGT	NM_017008
Gch1	GTGTATGGTCATGCGAGGTG	GGAACTCCTCCCGAGTCTTT	NM_024356
Gnrh1	CCCCAGAACTTCGAATGCAC	CTTCTTCTGCCCAGCTTCCT	NM_012767
Gsn	CAGAGGCTCTTCCAGGTCAA	GGAGCCACACCACTGATAGA	NM_001004080
Hand2	CAGCTACATCGCCTACCTCA	CTCCTCTTTCACGTCGGTCT	NM_022696
Hrh3	GCCACTGCTATGCTGAGTTC	CGCCTCTGGATGTTCAGGTA	NM_001270566
Ifi30	TCTCTGCACCACCTGTCAAT	GTAGGGCACCAGAGTGATGT	NM_001030026
Iqub	TTTCCTCAGAACCCGACCAA	CCACAGCTACCTCCTCGTAG	NM_001034130
Itgb5	CAATGAGTACACTGCGTCCG	GTTCCAGGTATCAGGGCTGT	NM_147139
Itgb8	GGAGGTTTTGATGCCATGCT	GCTGTCAAGAGCGAGATGTG	NM_001108726
Kcnj10	CTTTGGCGTGGTGTGGTATC	AAGGCCCCAAGGTAAGTGTG	NM_031602
Kcnk13	CAACAGGAAGGCCCCTAGAA	GATGGCTACTGATGCCAAGC	NM_022293
Klk8	GAGTGTAAACCCCACTCCCA	ACGCACGGAGTACTTGTCTT	NM_001107509
Met	AGTGTCCCGACTGTGTAGTG	ATCGGGAGGGTAGGAAGAGT	NM_031517
Mmp9	AAAGGTCGCTCGGATGGTTA	GGAAGACGCACATCTCTCCT	NM_031055
Mylpf	CGACGCCATGATGAAGGAAG	AGGCTCCAGTGATCACATCC	NM_016754
Nrl	TGAGTCCTGATGAGGCTGTG	CGGCGTCTGAAAATCTCTCG	NM_001106036
Nupr1	AGCCTGGCCCAATCTTATGT	GCCAGGCCTTTTTCCTTTCA	NM_053611
Pcdh8	GCTGATCGTCATCATCGTGT	GCACTGTAGGGCTCCTTCTT	NM_022868
Pik3r5	TTGCTTACCTCCTCTCAGGC	GGAGCTTTCCTCACTGTCCT	NM_001191923
Prtn3	TCCAGGCCTCCAAGATAGTG	ACGAATCTCGGGTGGATCAG	NM_001024264
Reln	GCGTCAATGGAACCAAGTGT	AGGTTTCCCACCGCTCATTA	NM_080394
Rxfp2	GAATGTTCCGGCCCATGAAG	ACCATCAGTCGAGGGCATAC	NM_001012475
Sdc4	AGGTCTTGGCAGCTCTGATT	CCCAAGTCGTAACTGCCTTC	NM_012649
Slc6a1	TCCCTGATTGCTCTGGGAAG	ATGAAGCCCACGATGGAGAA	NM_024371
Sphk1	GTATGCCCTCGACTGATGGT	CTCACGGCTATGACGAGGAA	NM_001270811
Vav2	TGACCCCATGCACAACAAAG	GCTCCATCCACTTCCGTTTC	NM_001106563
Wasl	TCTTTTCTCCTTCCTCGGCA	GCAACACCACTGCACTTCTT	NM_001110365

*Acc. No. indicates gene access number.
